# A need-driven design research of modular functional armchairs for young adults

**DOI:** 10.1038/s41598-026-49680-z

**Published:** 2026-04-29

**Authors:** Yanfeng Miao, Jia Li, Xuefei Gao, Wei Xu

**Affiliations:** 1 College of Furnishings and Industrial Design, Nanjing Forestry University, Nanjing, 210037 China; 2 Jiangsu Co-Innovation Center of Efficient Processing and Utilization of Forest Resources, Nanjing, China

**Keywords:** Functional armchair, Kano model, Modular design, Design evaluation, Engineering, Mathematics and computing

## Abstract

This study proposes a need-driven framework for the modular design of functional armchairs for young adults living alone. It aims to establish a systematic linkage among user-need priorities, functional organization, and product architecture generation. User needs were collected through observations, interviews, and questionnaires, leading to the identification of 15 core functions. These functions were transformed into 11 functional modules using function–structure mapping, correlation assessment, and hierarchical clustering. AHP was applied to determine evaluation weights, and TOPSIS was used to compare design alternatives. The results show that the optimized solution better aligns with user expectations in both functionality and experience. The study contributes a need-to-module translation mechanism that positions modularization as an intermediate decision layer between need prioritization and product implementation. This framework establishes a traceable pathway linking user needs, module generation, and design evaluation, offering methodological support for complex product design.

**Supplementary Information:**

The online version contains supplementary material available at 10.1038/s41598-026-49680-z.

## Introduction

With social development, lifestyle transformation^1^, and the continued growth of single-person households^2,3^, young adults living alone are showing increasing demand for home furnishings that combine comfort, flexibility^4,5^, and personalized functionality^6,7^. In this context, the functional armchair has evolved from a conventional seating product into an integrated household solution that can support leisure and work^1,8,9^. Against the backdrop of increasingly compact living spaces and continuously changing lifestyles, how product design can more effectively respond to the diversified needs of young adults living alone has become an important issue in both the research and practice of functional furniture design.

Despite this trend, the design of functional armchairs still faces two persistent challenges. First, user needs are becoming increasingly diverse and segmented, whereas conventional furniture design still relies heavily on designer experience and qualitative judgment^10^, resulting in a lack of systematicity in the processes of demand identification, screening, and translation. Second, in the pursuit of product differentiation, manufacturers often incorporate an increasing number of functions into a single product^11^. However, excessive feature addition may reduce post-purchase usability, induce feature fatigue^12^, and increase both structural complexity and development cost^13^. Accordingly, how to effectively integrate functions on the basis of accurately identifying and prioritizing user needs has become a key issue in functional armchair design.

Existing studies have provided important support for addressing this issue. Research on user needs has advanced the elicitation^14,15^, analysis, and categorization^16,17^ of user requirements, while studies on requirement translation have introduced tools for linking user demands with technical solutions^18,19^. Meanwhile, modular design has been widely recognized as an effective approach for improving customization^20^, maintainability, adaptability^21^, and production efficiency^22^ in product development. These studies have contributed valuable insights into either front-end demand analysis or back-end product realization. However, they have not sufficiently explained how prioritized user needs can be systematically translated into reproducible product architecture decisions, particularly in the design of multifunctional furniture. In particular, the connection between demand prioritization, functional integration, and module generation remains underdeveloped.

The Kano model is especially useful for front-end requirement screening^23^ because it helps distinguish between different categories of user needs and provides a basis for identifying which functions should be prioritized^24^. However, Kano analysis primarily addresses which functions matter most rather than how these functions should be structurally integrated into a product system. This distinction is especially important in functional armchair design, where multiple candidate functions often coexist. If each need is directly translated into an independent design feature, the result may be function proliferation, increased structural complexity, and higher development costs. Therefore, the Kano model is more suitable as a front-end demand screening mechanism rather than a complete design solution.

Modular design provides a feasible pathway for translating prioritized user needs into product realization. By decomposing and recombining product elements, modular design can support functional integration while improving flexibility and configurability^25^. However, existing studies on modularization have largely treated it as a means of achieving product flexibility^26,27^, while paying relatively limited attention to why and how different types of demand attributes should correspond to different modularization strategies. Accordingly, in this study, modular design is conceptualized as an intermediate decision-making layer linking demand priorities with product implementation logic, enabling different categories of user needs to be translated into actionable functional modules.

To address the above limitations, this study constructs a pathway from user needs to product architecture generation by integrating Kano-based demand analysis with modular design methods, with the aim of establishing a systematic linkage among user-need priorities, functional organization, and product architecture generation. First, the Kano model is employed to identify and classify the needs for functional armchairs intended for young adults living alone, distinguishing core, secondary, and optional functions so as to reduce functional redundancy during the demand-screening stage. Subsequently, modular design is introduced to integrate highly related core functions into shared structures or composite modules, while transforming lower-priority needs into detachable and configurable optional modules, thereby enhancing product flexibility and personalized adaptability while controlling complexity and cost.

Based on this, this paper takes functional armchairs for young adults living alone as a case study and proposes and validates a demand-driven modular design framework, with a focus on addressing the following research questions:

### RQ1

 How can the functional needs of young adults living alone be systematically identified and prioritized for functional armchair design?

### RQ2

 How can prioritized user requirements be translated into reproducible functional modules rather than directly into isolated design features?

### RQ3

 How can alternative design solutions be evaluated in a way that connects user needs, modular architecture, and overall design performance?

The contributions of this study are threefold. First, it establishes a need-driven design framework in which product function configuration is driven by the prioritization of user needs, thereby providing a more systematic and user-oriented approach to functional furniture design. Second, it reconceptualizes modularization as an intermediate decision layer linking demand priorities to product implementation logic, and proposes a stepwise translation pathway consisting of function–structure mapping, correlation evaluation, and hierarchical clustering, thereby enhancing the interpretability and reproducibility of the module generation process. Third, using functional armchairs for young adults living alone as a case study, this study validates the applicability of the proposed framework to the integrated design of complex functional furniture and also offers a reusable methodological reference for the design of other durable consumer products driven by user needs.

The remainder of this paper is organized as follows. Section “Literature review” reviews the relevant literature on user-needs research and modular design. Section “Methodology” presents the research framework and methods. Section “Case study” reports the case study and design evaluation process. Section “Discussion” discusses the findings, contributions, and limitations. Finally, Section “Conclusion” concludes the paper and outlines future research directions.

## Literature review

### User needs research

User needs are widely recognized as a central concern in product design because they shape product definition, user experience, and market competitiveness^28^. Existing studies generally agree that user-centered design requires designers to incorporate user needs throughout the product development process rather than treat them as an isolated preliminary task^29^. In the context of increasingly diversified consumption and the growing prevalence of smart products, the challenge is no longer merely how to collect user feedback, but how to transform heterogeneous user needs into actionable design knowledge and, ultimately, into product solutions. This issue is especially important for multifunctional products, whose design involves not only requirement identification but also functional integration and architectural coordination.

Existing research on user needs can be broadly discussed from three main directions. Fist, some studies focused on need elicitation. Traditional methods such as questionnaires, interviews, and focus groups remain important approaches for identifying explicit user expectations^30^. With the development of internet technologies, user-generated content, including online reviews and social media data, has become an additional and increasingly important source of need-related information^31,32^. Researchers have therefore introduced natural language processing^33^, big data analytics^34^, and artificial intelligence techniques^35,36^ to mine dynamic user feedback and predict emerging demands more efficiently. This direction has significantly expanded the scope and timeliness of user needs acquisition^37^.

The second direction concerns need modeling and prioritization. In this line of research, user needs are not only collected but also categorized, weighted, and interpreted^38^. The Kano model is widely used to distinguish between basic, performance, and excitement needs, thereby revealing the differentiated effects of product attributes on user satisfaction^39^. Some studies further integrate Kano with decision-making tools such as AHP or FCE to support need prioritization and design evaluation^40,41^. Compared with simple need collection, this direction provides a more structured understanding of what users value and why certain requirements should be given priority in product development.

Other studies address the translation of needs into technical or design solutions. Methods such as QFD^42^, FBS^43^, DSM, and related frameworks attempt to establish mappings between user requirements, product functions, and structural elements^44,45^. These studies have contributed important tools for connecting customer requirements with engineering decisions, and they are particularly valuable in complex product development where multiple functions and structural relationships must be coordinated^46^. In this sense, prior research has already moved beyond need identification toward the problem of requirement implementation.

Despite these advances, important limitations remain. First, studies on need elicitation and prioritization are effective in clarifying what users value most, but they often stop at the level of requirement identification and ranking. As a result, they provide limited explanation of how differentiated categories of user needs can be translated into product architecture. Second, studies on need-to-technical translation offer useful mapping tools, but they tend to treat user needs as relatively uniform inputs and pay insufficient attention to how different need attributes should lead to different strategies of functional integration and module partitioning, particularly in the design of multifunctional furniture. Third, ambiguity in need expression and the possibility of designer misinterpretation may weaken the reliability of the transition from user needs to functional and structural specifications^47^.

Therefore, the key gap in the existing literature is not simply the absence of methods for collecting or translating needs, but the lack of an integrated logic that connects need categorization, functional integration, module generation, and design evaluation. To address this gap, the present study does not use the Kano model merely as a descriptive tool for classifying user needs. Instead, it repositions Kano as a front-end decision mechanism through which differentiated need categories inform subsequent functional integration, module generation, and solution evaluation in multifunctional furniture design. In this way, the study extends prior user-needs research from requirement identification toward an interpretable design pathway linking user demand to product architecture.

### Modular design

Modular design is generally understood as a design approach in which a product is decomposed into relatively independent but connectable units based on functional analysis and usage characteristics, and these units are subsequently combined through standardized interfaces to form a complete product. As an important design strategy in modern manufacturing, modular design has been widely adopted because it supports product variety, production efficiency, and system flexibility^48^. For multifunctional products in particular, modular thinking offers a practical way to coordinate diverse functions while maintaining adaptability and manufacturability^49^.

Existing studies on modular design mainly develop along two directions. The first direction emphasizes the practical value of modularity in terms of customization, flexibility, and production efficiency. By decomposing products into interchangeable modules, designers can generate a wider range of product variants to satisfy personalized market demands^50^, while also reducing development time and manufacturing costs through parallel production and standardized interfaces^51^. In furniture design, modularity has been associated with flexibility^52^, multifunctionality^20^, transport convenience^53^, and improved adaptability to different living spaces^54^. This direction demonstrates why modular design is attractive from both user and manufacturing perspectives.

The second direction focuses on methods for modular partitioning and architecture generation. In this line of work, tools such as DSM, QFD, and MFD are used to analyze relationships among functions, components, and technical requirements in order to derive more systematic module structures^55,56^. Compared with purely experience-based design, these methods improve the rigor of module generation and provide a more explicit basis for handling complex product systems. In some studies, MFD is combined with DSM to connect customer requirements with functional and technical solutions, thereby supporting the derivation of modular architectures^57^.

However, existing modular design research still exhibits several limitations when applied to multifunctional furniture. First, in many studies, module partitioning is still strongly influenced by designers’ subjective judgment, even when the stated goal is personalization or flexible configuration. As a result, the process by which functions are integrated and modules are defined is not always sufficiently explicit or reproducible. Second, although some studies adopt quantitative methods, modularization is often treated primarily as a problem of structural decomposition. In such cases, modular design appears mainly as an implementation outcome rather than as a decision layer that mediates between user needs and product realization. Consequently, the literature does not yet adequately explain how different categories of user needs should lead to differentiated module strategies in multifunctional products.

From this perspective, the theoretical limitation of existing modular design research lies not in the absence of modular methods, but in the insufficient conceptualization of what modularization does in the overall design logic. In response, the present study conceptualizes modularization as an intermediate decision layer between user needs and product implementation. Rather than viewing modularization merely as structural decomposition, this study interprets it as the mechanism through which differentiated user needs are translated into functional integration strategies, module partitioning decisions, and ultimately evaluable design solutions. This reconceptualization extends existing modular design theory by clarifying the role of modularization in connecting user demand analysis with product architecture generation, thereby improving the interpretability and reproducibility of multifunctional furniture design.

## Methodology

### Proposed framework

This study adopts a sequential methodological framework. First, the Kano model is used to classify and prioritize user requirements, thereby determining whether specific functions should be integrated into core modules or configured as detachable optional modules. Second, modularization is positioned as an intermediate decision layer that translates prioritized requirements into implementable functional modules through function–structure mapping, correlation assessment, and hierarchical clustering. Third, after the module architecture has been generated and embodied in design concepts, AHP is used to derive the relative importance of the evaluation criteria, while TOPSIS is employed to compare alternative design schemes against a benchmark solution.

**Fig. 1:**
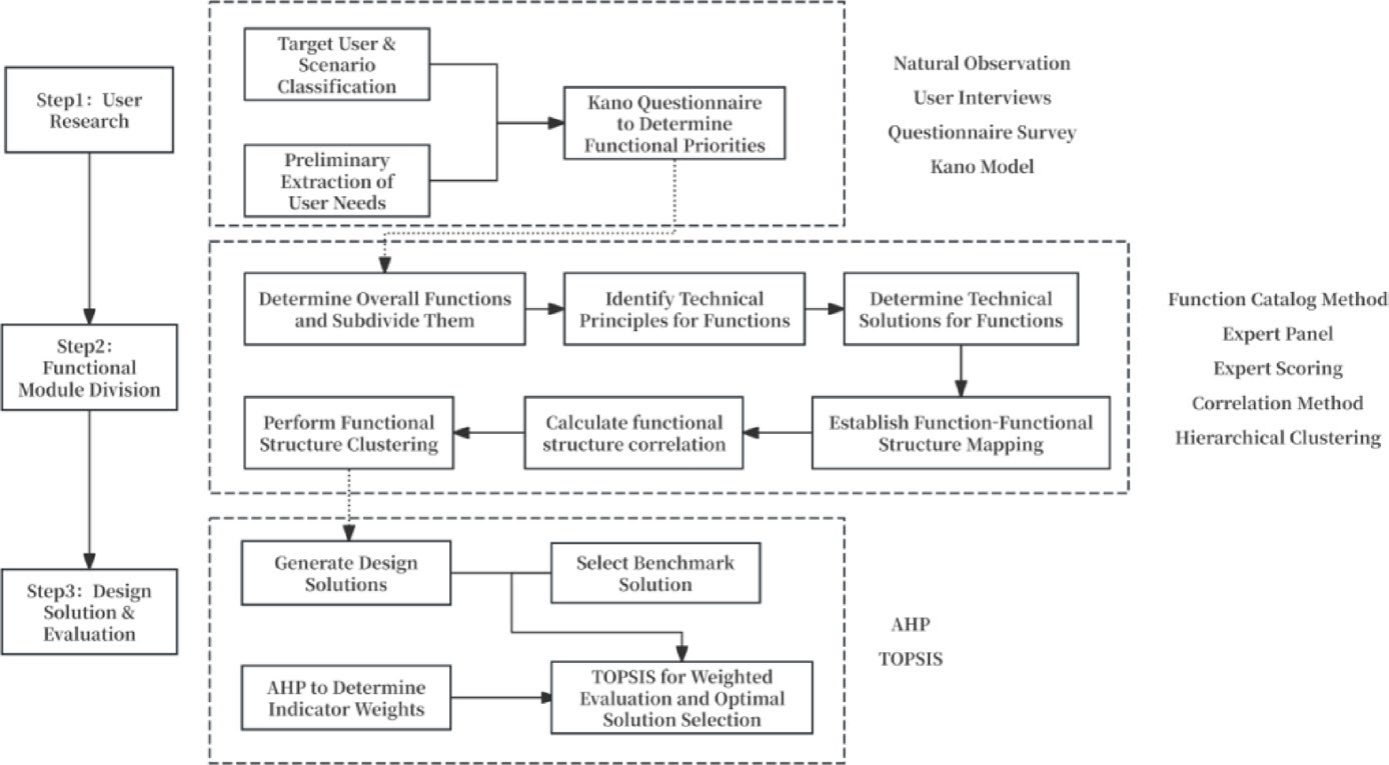
Technical roadmap.

The theoretical novelty of this framework lies in articulating an explicit translation mechanism from requirement prioritization to module architecture generation and subsequent design evaluation. Rather than treating modular design as a tool for structural decomposition alone, this study reconceptualizes it as the critical link between differentiated user requirements and product implementation logic. In doing so, the framework makes the transition from user requirements to module boundaries more explicit, traceable, and reproducible, while also establishing a closed-loop linkage in which design schemes are evaluated against criteria derived from the same requirement structure that informed their generation. Figure 1 presents the technical roadmap of the proposed framework.

### Kano model

The primary objective of the Kano model is to thoroughly investigate user needs, classify and prioritize them, and evaluate user satisfaction levels with specific product attributes^58^. Based on the impact of user requirements on satisfaction, it reflects the nonlinear relationship between product performance and user satisfaction^59^.

In this study, a Kano questionnaire was designed to investigate the user needs of young adults living alone regarding functional armchairs. The aims were to classify these needs, clarify their priorities, and thereby ensure the accuracy and completeness of requirement identification, enhancing the scientific rigor and objectivity of design decisions.

### Modular design

While product function integration enhances product competitiveness, excessive functional incorporation often leads to reduced production efficiency. Using a modular approach to function integration can significantly shorten the production cycle of integrated products. On the other hand, non-essential functions should be designed as detachable modular accessories to accommodate diverse user needs. Therefore, this section explores the functional design of the armchair using a modular design methodology, and the following modular design process has been developed in this study:

1. Determine the overall function of the design object and break down the core functions into sub-functions.
2. Identify technical principles for achieving each function.
3. Define technical solutions for function implementation.
4. Establish mapping relationships between functions and functional structures.
5. Calculate the correlation degrees between functional structures.
6. Perform clustering analysis of functional structures.

In this study, the function catalog method will be employed to classify and organize potential solutions and determine technical solutions for function implementation. Due to the interrelationships between functional requirements and basic functional modules, these modules are further decomposed into sub-level functional units or functional structures, which serve as the basis for subsequent module integration^60^. The concept of correlation degree is introduced to quantify the interdependence between functional structures. Experts are invited to score these correlations, after which an evaluation system is applied to calculate the weighted overall correlation. Finally, hierarchical clustering is used to progressively group highly correlated functions, forming multiple sets of functional modules^61^.

### AHP

The Analytic Hierarchy Process (AHP) can hierarchically structure complex decision-making problems, providing a scientific basis for quantifying evaluation metrics^62^. It comprises elements such as the goal layer, criterion layer, and indicator layer. By performing pairwise comparisons of various indicators and their underlying factors under specific requirements, numerical assignments are determined to identify the optimal design elements and importance weight factors for design solutions^63^.

A judgment matrix for the criterion layer and sub-criterion layer indicators is constructed and subjected to a consistency check. All expert evaluation data that passed the consistency test are integrated and comprehensively processed using the geometric mean method, ultimately forming a global judgment matrix. This comprehensive judgment matrix is then normalized, and the online statistical software “SPSSAU” is employed to calculate the weight values of each indicator relative to the objectives at each hierarchical level using the sum-product method. However, AHP still involves subjective decision-making, and the resulting weights may be influenced by the evaluators’ background and preferences. Therefore, the weights obtained in this study should be understood as context-specific rather than universally applicable.

### TOPSIS

The 16 evaluation indicators derived from the AHP method were used as assessment criteria in the TOPSIS approach, all of which are positive indicators. Experts and users were invited to jointly score the design alternatives, evaluating the extent to which each design meets user needs. Ratings were collected using a seven-point Likert scale (where 1 to 7 respectively represent: very poor, poor, slightly poor, neutral, good, very good, excellent). The evaluation process followed the computational procedures of TOPSIS, including the normalization of the evaluation matrix, construction of the weighted standardized matrix, determination of the positive and negative ideal solutions, and calculation of the relative closeness, to decide on the best functional armchair design for young adults^64^.

To ensure objectivity in the design alternative selection process, a focus group method was first employed to select the best-performing armchair product from a pool of options summarized during the preliminary market research phase. The selection was based on comprehensive performance across functionality, materials, form, and other dimensions. This product served as the benchmark sample for evaluation. Scores from experts and users for both the design alternatives and the benchmark sample were averaged to form the initial evaluation matrix *F*. Nevertheless, averaging expert and user scores may conceal differences between the two groups. Therefore, the TOPSIS results should be interpreted as a comparative evaluation within this case study.

## Case study

### User needs research for functional armchair

#### User Survey

To accurately acquire user needs, this study targeted young adults aged 25–34 living alone, employing a mixed-methods approach integrating observation, interviews, and a Kano questionnaire. The investigation aimed to: (1) construct user profiles; (2) analyze their activity patterns based on sofa usage; (3) identify dissatisfactions with existing products; and (4) explore their functional expectations for functional armchairs. Through this process, the needs were systematically screened and prioritized.

Numerous studies have utilized video materials from the YouTube platform as a data source for related research^65^. Short videos on these platforms can intuitively illustrate users’ behavioral processes, postural patterns, and environmental characteristics in natural settings, thereby assisting researchers in identifying user needs from a dynamic perspective^66^. Based on this rationale, short videos were selected from various platforms as observation samples to inductively analyze users’ behavioral manifestations and daily contexts when utilizing functional sofas.

This study initially conducted contextual observations of short videos depicting home life on platforms such as Bilibili, Douyin, and Xiaohongshu. The search keywords were predefined as “living alone,” “daily home life,” “sofa/armchair usage,” “working from home,” and “watching series/gaming/reading at home.” The search timeframe was restricted to publicly available videos published within the past 24 months. The initial search yielded a total sample of 134 videos. Prior to formal analysis, these videos underwent a screening process; the specific screening criteria and procedures are presented in Table 1.

**Table 1:** Filtering process of video samples.

No.	Exclusion criteria	Excluded videos	Remaining videos
1	Publisher aged 25–34; confirmed self-posted and self-appearing; content clearly shows a single-person home setting	17	117
2	Shows continuous real-life behavioral clips related to seating, rather than just product displays or scripted montages	22	95
3	Videos with unclear information or weak relevance to the research topic	9	86
4	Videos with complete audio/visual info and sufficient length to identify postures, behaviors, contexts, and objects involved	26	60

**Fig. 2:**
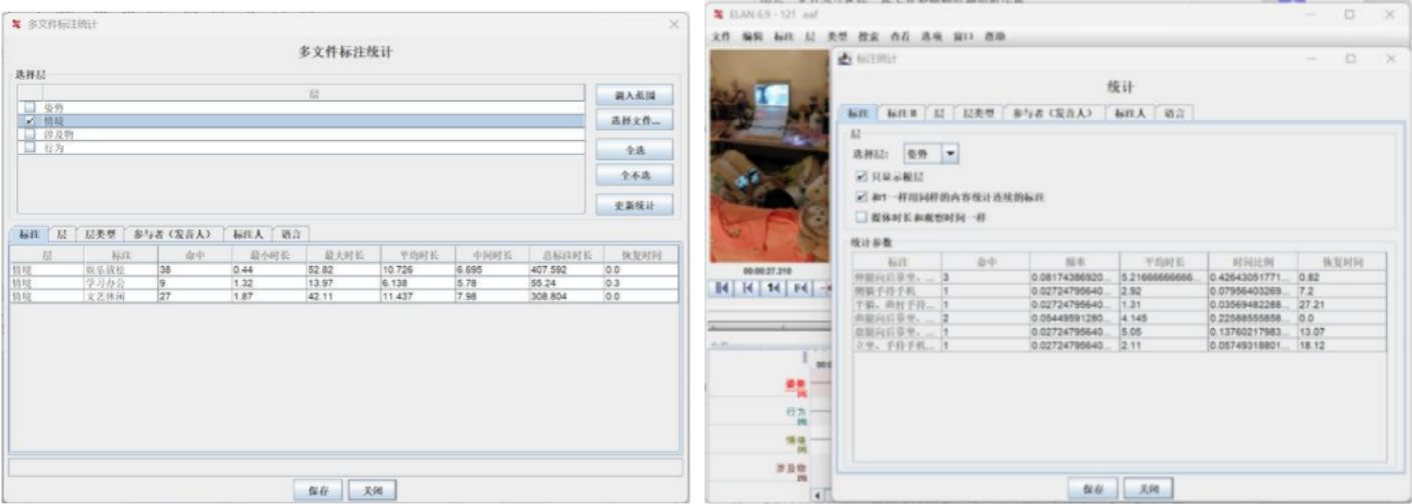
ELAN video annotation diagram.

Ultimately, 60 representative videos were selected, and ELAN version 6.9 was employed to annotate and statistically analyze the screened video data. In this study, the annotation tiers were categorized into posture, behavior, context, and interacting objects, as illustrated in Fig. 2.

The behavioral activities of the observed subjects were statistically analyzed. Based on behavioral characteristics, the home-based lifestyle of the target group and their contexts of interaction with sofas can be categorized into the following three types: Study and Work (9 occurrences), Cultural and Recreational Activities (27 occurrences), and Leisure and Relaxation (38 occurrences), as summarized in Table 2.   

**Figure:**
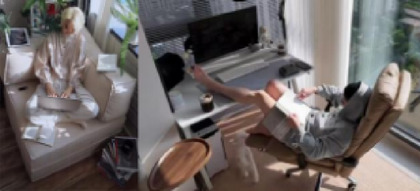

**Figure:**
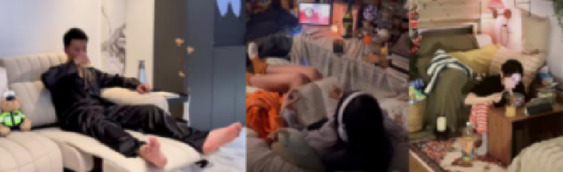

**Figure:**
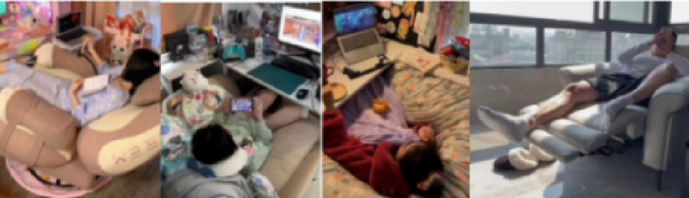

This study employed semi-structured interviews combined with the laddering technique to gain in-depth insights into the daily activities at home, sofa usage patterns, and specific needs regarding functional armchairs among young adults. Participant screening criteria required individuals to be aged 25–34, possess a stable income, currently live alone, and spend an average of more than two hours per day on a sofa. Prior to the interviews, participants were briefed on the concept of functional armchairs, including their modular functionalities and market price range. The detailed interview protocol is presented in Table 3.

**Table 3:** Interview protocol.

Number: Date:	Sex: Age:	Occupation: Dwelling state:
Personal information	What is your consumption concept? What do you think of multi-functional products/sofas?…	
Home behaviour habits	Do you prefer to stay in bed or on the sofa when you rest? Why? What do you usually do (in what posture) on the couch? How long can you stay on the couch?…	
Usage details of sofa products	What are the discomforts/conveniences you experience when you spend a long time on the couch? Which functions do you think are necessary and which are not necessary for the functional armchair on the market at present? (Explain one by one with the function description table)…	
Functional armchair expectation concept	What features would you like to add to a functional armchair if there were no restrictions? Why?…	

In total, nine employed young adults living alone were interviewed, with each session lasting between 20 and 30 min. Detailed respondent information is presented in Table 4. By the seventh interview, feedback regarding functional sofa requirements had become highly repetitive, and upon completing the ninth interview, no new core functional expectations or pain points emerged. This indicated that data saturation was reached, confirming that the sample size of nine users was sufficient to support the requirements of the study’s qualitative analysis.

**Table 4:** Information of respondents.

ID	Gender	Age	Education	Occupation	Housing Status
B1	Female	26	Bachelor’s	Furniture Designer	Homeowner
B2	Male	28	Bachelor’s	Designer	Long-term Rental
B3	Female	26	Master’s	Product Planning Specialist	Homeowner
B4	Female	27	Bachelor’s	Structural Engineer	Long-term Rental
B5	Male	28	Master’s	Product Designer	Long-term Rental
B6	Female	32	Bachelor’s	Marketing Manager	Long-term Rental
B7	Male	29	Master’s	Product Manager	Long-term Rental
B8	Female	29	Master’s	Product Designer	Long-term Rental
B9	Male	29	Bachelor’s	Product Manager	Long-term Rental

Interviews with nine employed young adults living alone revealed an average daily sofa usage time exceeding two hours, with primary activities concentrated on smartphone use, remote work, video streaming, and gaming. Based on these high-frequency usage scenarios, users commonly expressed strong demand for integrated “small tabletop/storage surface” and “ventilation/breathability” functions. The former need particularly stems from work-from-home habits and remains inadequately addressed by current market offerings.

The nine respondents evaluated the functions of existing functional armchairs available on the market. Their assessments regarding “essential” features were statistically analyzed and summarized in Table 5, where “√” indicates “Essential” and “○” indicates “Non-essential.”

**Table 5:** Functional evaluation and requirement statistics.

	Sit-to-lie conversion	Forward-and-backward gentle rocking	Left-and-right swiveling	Headrest adjustment	Lumbar support adjustment	Armrest height adjustment	Charging interface	Storage	Phone holder	Wireless phone charging	Wireless power storage	Backrest massage	Seat cushion heating	Voice control	APP control	Bluetooth speaker	Assisted lifting	Seat height adjustment
B1	√	○	√	√	√	√	○	○	○	○	√	√	○	○	○	○	√	○
B2	√	√	√	√	√	√	○	○	√	√	√	√	○	○	√	○	√	√
B3	√	○	○	√	√	√	√	○	○	○	○	√	√	○	○	○	○	○
B4	√	○	○	√	√	√	○	√	√	○	○	√	○	○	○	○	○	○
B5	√	√	○	√	√	√	○	○	○	○	○	√	√	√	√	○	√	√
B6	√	○	○	√	√	√	√	○	○	√	√	√	√	○	○	○	○	○
B7	√	√	√	√	√	√	○	○	○	○	○	√	√	○	○	○	○	○
B8	√	○	○	√	√	√	√	√	○	√	○	√	○	√	√	√	○	○
B9	√	○	√	√	√	√	√	○	√	√	○	√	○	○	○	○	○	○
Total	9	3	4	9	9	9	4	2	3	3	3	9	4	2	3	1	3	2

#### User requirement analysis

Based on the interview findings, five essential functions were identified: sit-to-lie conversion, headrest adjustment, lumbar support adjustment, armrest adjustment, and backrest massage. Consequently, these five items were excluded from the initial 18 functions, and five new functions—small tabletop, reading light, throw pillow, ventilation/breathability, and aroma diffusion—were added to formulate the Kano model questionnaire. Subsequently, an online questionnaire (as shown in Table 6) was distributed to young adults aged 25–34 living alone, resulting in 194 valid responses, to validate their attributes and priorities.

**Table 6:**
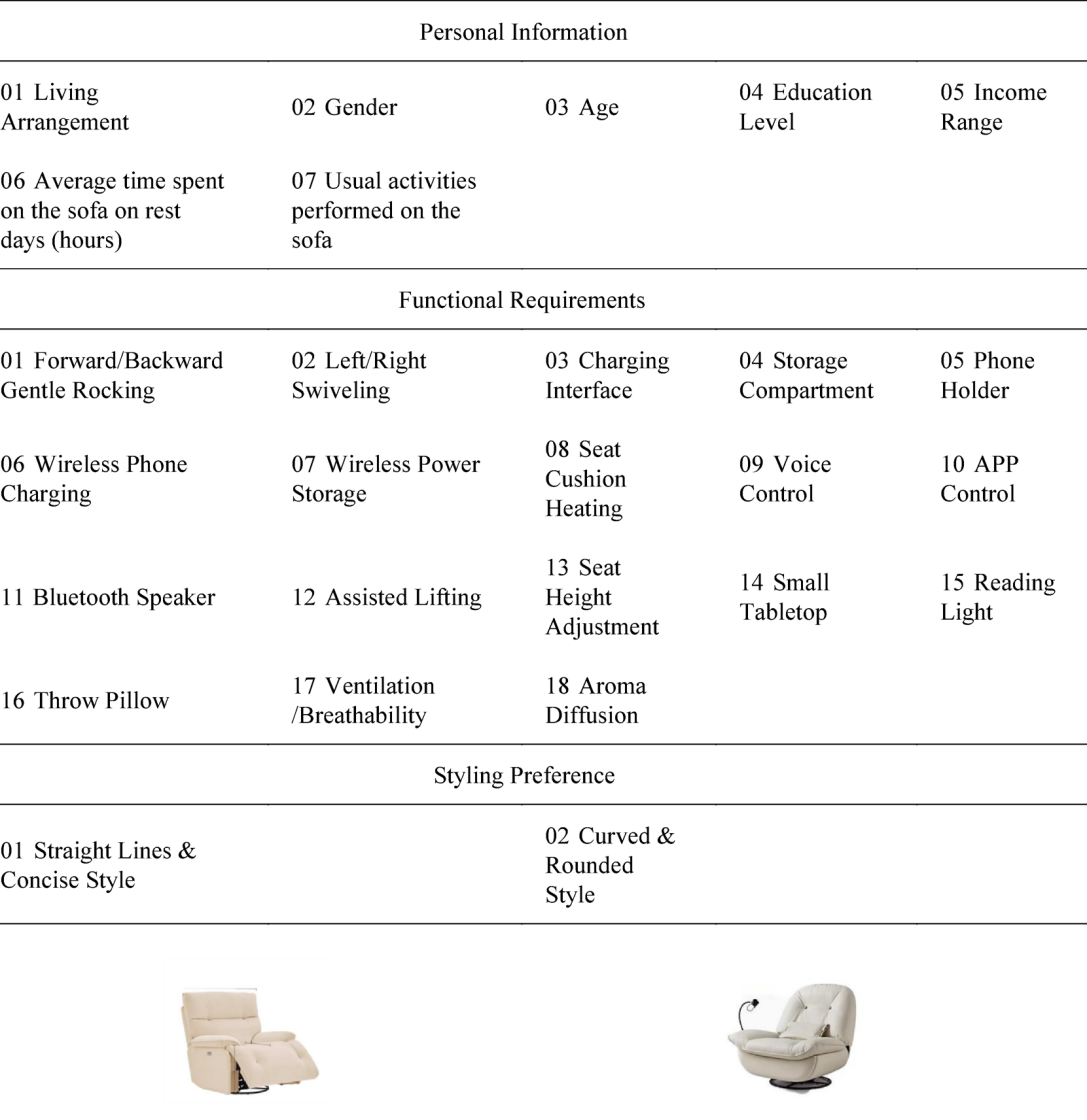
Questions in the survey questionnaire.

Reliability and validity analyses confirmed the questionnaire data’s robustness (Cronbach’s α > 0.7, KMO = 0.777, *p* < 0.05). The functional requirements of the armchair were statistically analyzed by calculating and comparing the proportion of each attribute category, with the category exhibiting the highest proportion designated as the primary attribute for each functional requirement. Simultaneously, the Better-Worse coefficient for each function was calculated, with the statistical results presented in Table 7.

**Table 7:** Statistics of functional requirement attributes.

Requirement	A	O	M	I	*R*	Q	Better	Worse	Attribute
Charging Port	0.144	0.021	0.577	0.227	0.031	0.000	0.170	0.617	M
Folding Tray Table	0.273	0.119	0.485	0.098	0.026	0.000	0.402	0.619	M
Ventilation	0.216	0.093	0.624	0.067	0.000	0.000	0.309	0.716	M
Wireless Power Bank	0.103	0.392	0.201	0.253	0.052	0.000	0.522	0.625	O
Seat Cushion Heating	0.093	0.510	0.108	0.227	0.062	0.000	0.643	0.659	O
Swivel Left/Right	0.340	0.134	0.180	0.304	0.041	0.000	0.495	0.328	A
Storage	0.392	0.113	0.160	0.294	0.041	0.000	0.527	0.285	A
Wireless Phone Charging	0.521	0.196	0.082	0.191	0.010	0.000	0.724	0.281	A
Reading Light	0.381	0.242	0.124	0.201	0.052	0.000	0.658	0.386	A
Hug Pillow	0.515	0.180	0.144	0.088	0.072	0.000	0.750	0.350	A
Gentle Rocking (Front/Back)	0.268	0.000	0.082	0.634	0.015	0.000	0.272	0.084	I
Phone Holder	0.345	0.103	0.108	0.428	0.015	0.000	0.455	0.215	I
Voice Control	0.263	0.170	0.160	0.392	0.015	0.000	0.440	0.335	I
APP Control	0.325	0.088	0.093	0.454	0.041	0.000	0.430	0.188	I
Assist to Stand	0.273	0.098	0.077	0.536	0.015	0.000	0.377	0.178	I
Seat Height Adjustment	0.211	0.119	0.149	0.510	0.010	0.000	0.333	0.271	I
Bluetooth Speaker	0.160	0.129	0.082	0.325	0.304	1.000	0.415	0.304	I
Scent Release	0.129	0.093	0.062	0.407	0.309	0.000	0.321	0.224	I

Kano model analysis classified the 18 user requirements into 3 Must-be requirements (M), 2 One-dimensional requirements (O), 5 Attractive requirements (A), and 8 Indifferent requirements (I). Consequently, eight indifferent requirements were excluded from this study. By integrating the five core must-be requirements identified through interviews, 15 functions requiring focused innovation or optimization were ultimately selected. These functions were further detailed (Table 8), with non-essential requirements designed as detachable modular accessories to enhance product flexibility and market adaptability.

**Table 8:** Functional armchair for young adults with functional configurations.

Functional category	Function	Sub-function	Functional description
Posture & Comfort Adjustment	F1. Sit-to-Lie Conversion	Multi-level Footrest/Backrest Adjustment	Precise automatic adjustment
		Comfortable Support in Sitting/Lying Positions	Actively conforms to the user’s body
		Pet Anti-pinch Function	Footrest stops automatically upon encountering an obstacle during downward adjustment
	F2. Headrest Adjustment	Multi-level Headrest Adjustment	Adjustable to different angles
	F3. Lumbar Support Adjustment	Multi-level Lumbar Support Adjustment	Adjustable to different angles
	F4. Armrest Height Adjustment	Multi-level Armrest Adjustment	Supports arms during smartphone use
	F5. Swivel Function	Rotation Lock	Swivels driven by user’s own force
Additional Convenience Functions	F6. Small Tabletop	Modular Connection	Easy to detach
		Object Placement	Holds items via the table surface
		Free Adjustment	Flexible adjustment by the user for convenient use
	F7. Reading Light	Modular Connection	Easy to detach
		Illumination	Provides lighting via LED lamp
		Automatic Induction	Automatically senses ambient light and human proximity to provide illumination
	F8. Storage	Modular Connection	Easy to detach
		Compartmentalized Storage	Designated sections for different stored items
	F9. Wireless Phone Charging	Modular Connection	Easy to detach
		Wireless Charging	Supports wireless charging for phones
	F10. Throw Pillow	Multi-purpose Pillow	Functions as both a throw pillow and a blanket
	F11. Wireless Power Storage	Wireless Power Storage	Can be used after being charged
	F12. Charging Interface	Charging via Interface	Interface compatible with different device models
Health & Relaxation Functions	F13. Backrest Massage	Multi-level Massage Adjustment	Provides different massage intensities
	F14. Seat Cushion Heating	Multi-level Heating Adjustment	Provides different heating temperatures
	F15. Ventilation	Multi-level Ventilation Adjustment	Provides different airflow speeds

### Functional module division for the youth functional armchair

#### Function catalog establishment

Through data collection and organization, this study established a function catalog for the 15 overall functions and their subdivided functional elements, with partial results shown in Table 9.

**Table 9:**
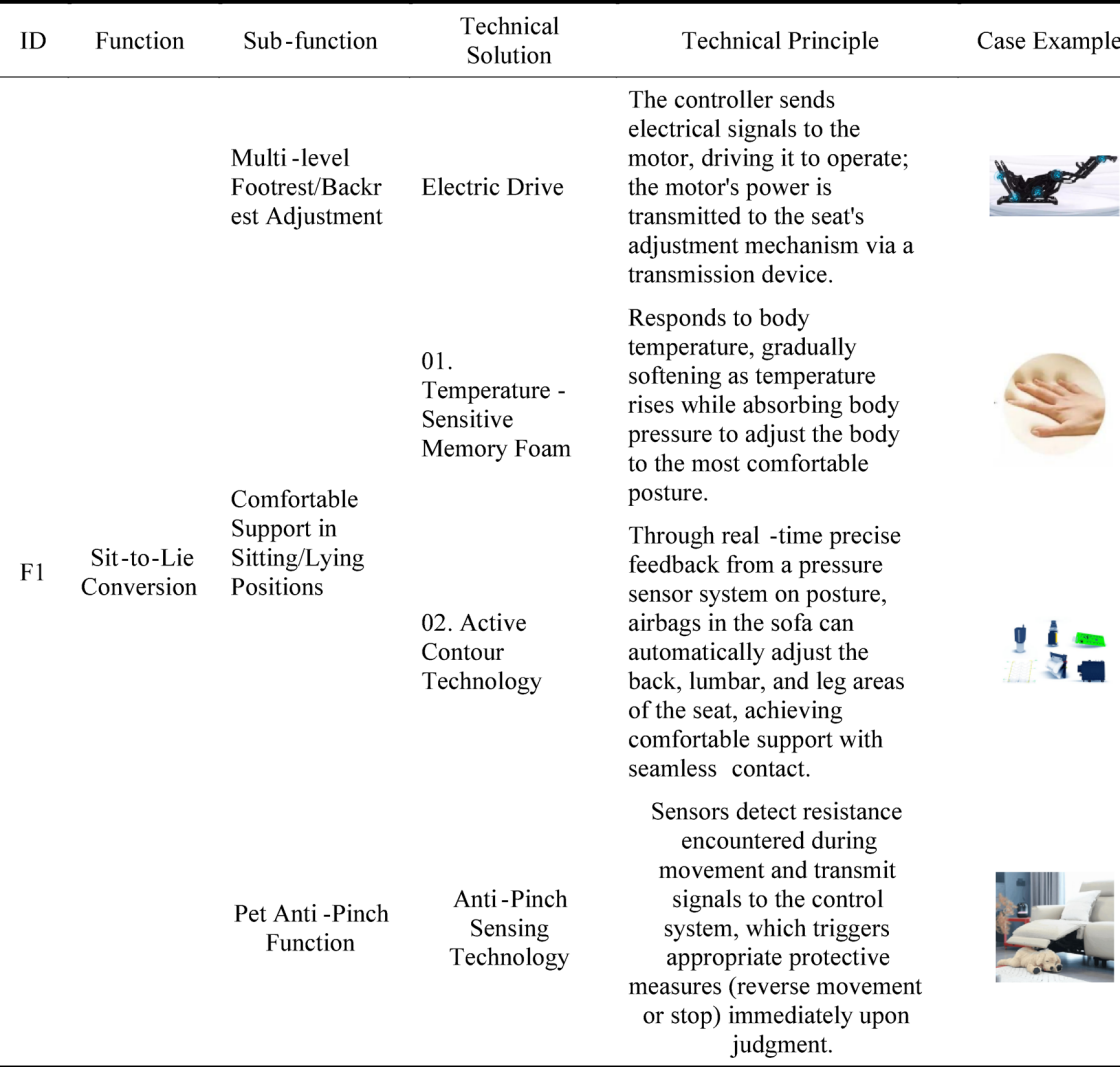
Function directory (portion).

#### Functional principle solution

To identify the most appropriate technical solutions for each functional requirement, an expert panel was established. The experts were selected based on the following criteria: (1) possessing a research background in furniture design or being engaged in furniture design practice; and (2) having at least two years of professional experience or research involvement in related fields.

The expert panel consisted of two upholstered furniture designers, two furniture structural engineers, and two home product planners, ensuring a balance between design theory and practical application within the design process. Previous studies^67^ have indicated that even with a relatively small expert sample, reliable judgments can still be achieved when the expertise and research background are well aligned.

Through a systematic evaluation and screening process, the most suitable technical solutions were determined for all 15 overall functions and their corresponding sub-functions. A summary of these specific solutions is presented in Table 10.

**Table 10:** Technical solutions for the new product.

Overall function code	Function	Sub-function code	Sub-function	Technical solution
F1	Sit-to-Lie Conversion	f1	Multi-level Footrest/Backrest Adjustment	Electric Drive
		f2	Comfortable Support in Sitting/Lying Positions	Active Contour Technology
		f3	Pet Anti-pinch Function	Anti-pinch Sensing Technology
F2	Headrest Adjustment	f4	Multi-level Headrest Adjustment	Pneumatic Headrest Adjustment
F3	Lumbar Support Adjustment	f5	Multi-level Lumbar Support Adjustment	Pneumatic Lumbar Support Adjustment
F4	Armrest Height Adjustment	f6	Multi-level Armrest Adjustment	Mechanical Adjustment and Locking
F5	Swivel Function	f7	Rotation Lock	Base Turntable + Mechanical Locking
F6	Small Tabletop	f8	Modular Connection	Traditional Mechanical Connection
		f9	Object Placement	Tabletop Panel
		f10	Free Adjustment	Mechanical Manual Adjustment
F7	Reading Light	f11	Modular Connection	Magnetic Connection
		f12	Illumination	LED Light
		f13	Automatic Induction	Light Sensing + Human Presence Sensing
F8	Storage	f14	Modular Connection	Magnetic Connection
		f15	Compartmentalized Storage	Designated Storage Compartments
F9	Wireless Phone Charging	f16	Modular Connection	Magnetic Connection
		f17	Wireless Charging	Electromagnetic Induction Technology
F10	Throw Pillow	f18	Multi-purpose	Folding Design + Button/Zipper Fastening
F11	Wireless Power Storage	f19	Wireless Power Storage	Lithium Battery Power Storage
F12	Charging Interface	f20	Wired Charging	Power Transmission + Interface Conversion
F13	Backrest Massage	f21	Multi-level Massage Adjustment	Pneumatic Massage
F14	Seat Cushion Heating	f22	Multi-level Heating Adjustment	Built-in Heating Element
F15	Ventilation	f23	Multi-level Ventilation Adjustment	

#### Establishing the function-function structure mapping model

**Fig. 3:**
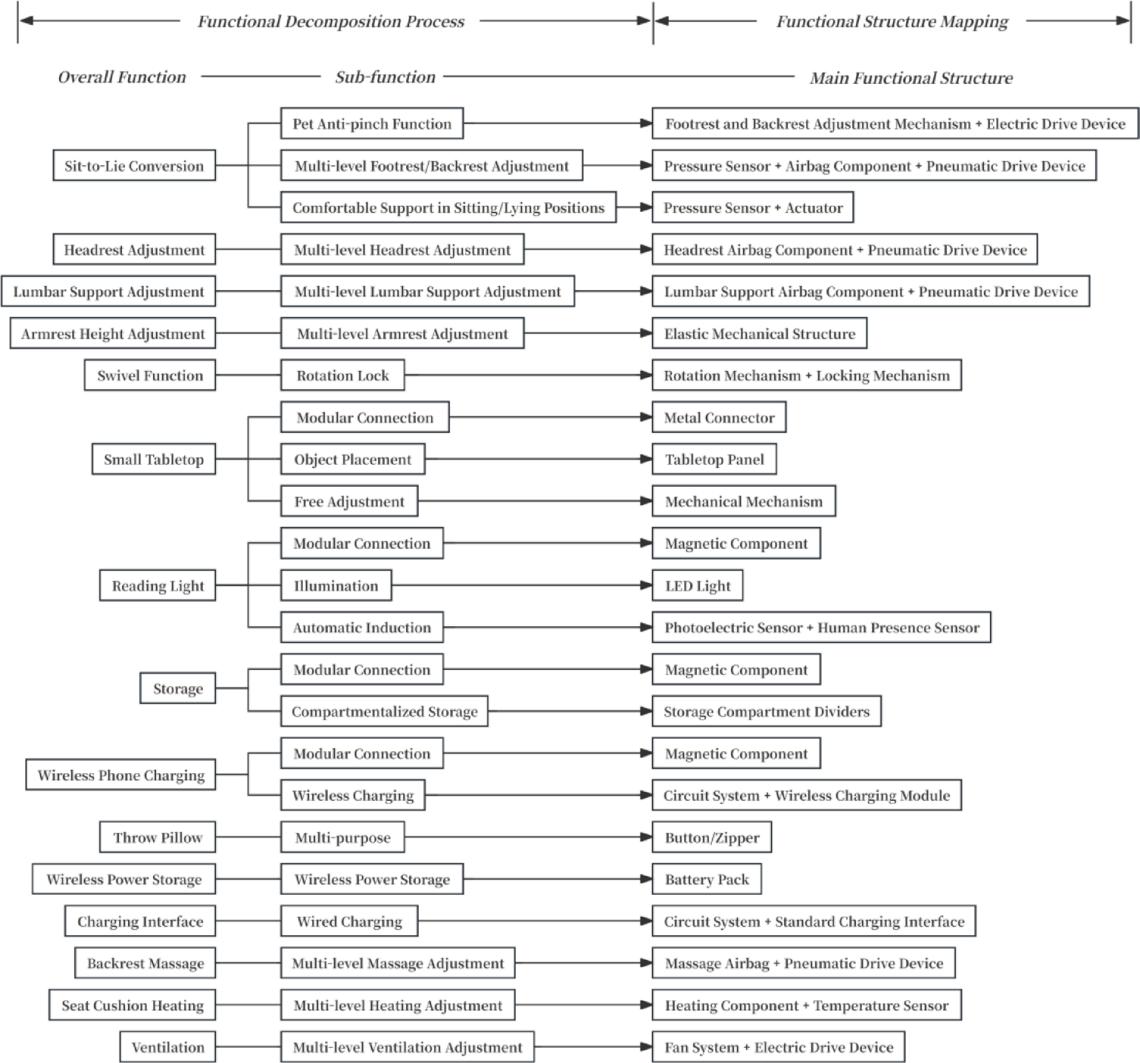
Functional and functional structure mapping model.

Based on the completed function catalog, each function or sub-function was mapped to its corresponding structural elements or components. This process established the mapping relationship between functions and functional structures, laying the groundwork for the subsequent correlation scoring phase. It required careful consideration of both the feasibility of function implementation and the rationality of the structural design. The mapping model between the functions and functional structure components of the target product is illustrated in Fig. 3.

#### Results of functional structure correlation

The expert panel established during the phase of determining the functional technical principles was invited to evaluate the importance of the three indicators using the nine-point scale method. Their weights were subsequently determined through the arithmetic mean. After passing the consistency check, the weight results are presented in Table 11. When assessing the degree of functional correlation, the expert panel fully considered practical usage scenarios within a living room context, incorporating layout factors such as the placement of side tables, the direction of reading light illumination, and the accessibility of storage and charging modules.

**Table 11:** Weight of correlation indicators.

Criterion	Weight	CR
Function	0.4083	0.0000 < 0.1000
Functional-Spatial Structure	0.5917	

**Fig. 4:**
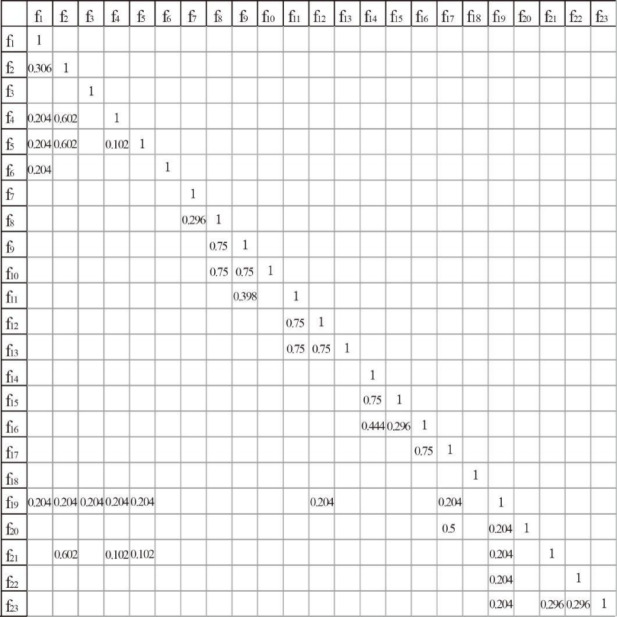
Functional structure relevance matrix.

The functional structure correlation matrix R_n_, determined by weighted calculation according to the formula, is shown in Fig. 4. The scoring matrices for functional correlation and functional-spatial structural correlation are provided in Appendix 2.

#### Results of functional structure clustering

**Fig. 5:**
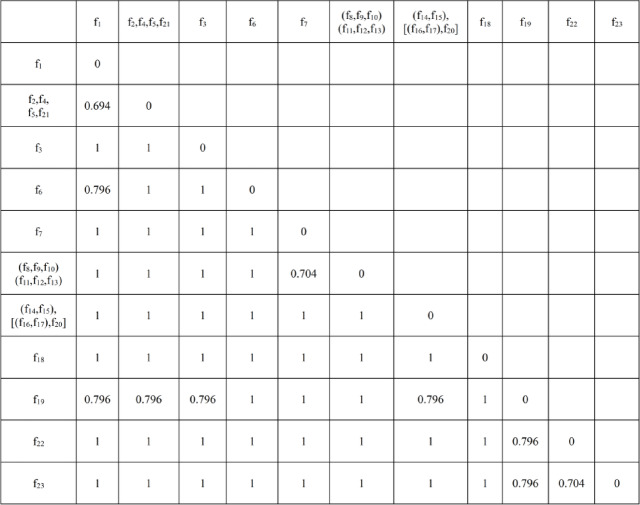
Final distance calculation results.

Comprehensively considering multiple factors such as user needs, production costs, and technical requirements for the target product, a module division threshold of 0.650 was selected for this study. The functional structure distance matrix D₀ underwent 4 iterative clustering cycles, resulting in a minimum association distance of 0.694. Since this value exceeds the threshold of 0.650, the clustering process was terminated. The final clustering result is depicted in Fig. 5.

The calculated values for the minimum association distance are listed in Table 12.

**Table 12:** Minimum correlation distance of functional structure.

Distance Matrix ID	D0	D1	D2	D3	D4
Minimum Distance Value	0.250	0.398	0.500	0.556	0.694

The final functional distance matrix represents the ultimate clustering outcome, where the 23 sub-functions are grouped into 11 functional modules through clustering. The division of functional modules in this study adheres to the principle of morphology-function co-optimization:

1. Based on morphological correlation, multi-airbag cooperative adjustment systems for the head, back, and lumbar regions were merged into a Biomechanical Support Module.
2. According to functional coupling, the charging interface and wireless charging system were integrated into a Human-Machine Interaction Power Supply Module.
3. Through the principle of spatial symbiosis, the magnetically integrated wireless charging device and the storage unit were combined to form a Spatially Adaptive Functional Module.
4. Based on environmental synergy, the reading light-small tabletop composite system was planned as a Healthy Office Support Module.

**Fig. 6:**
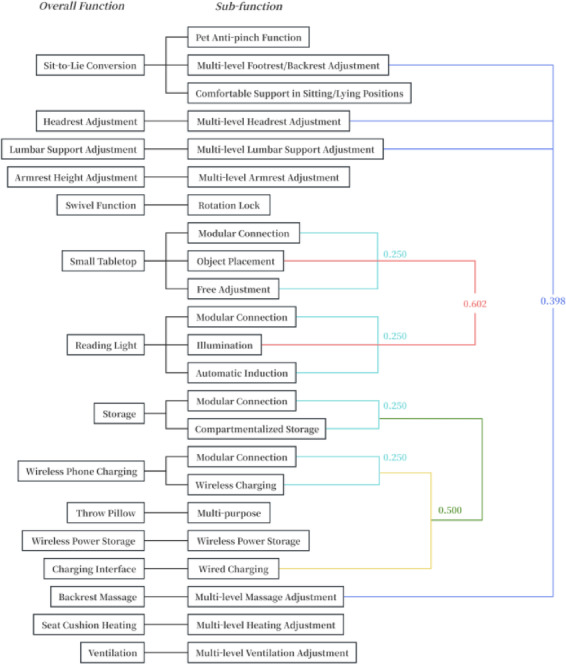
Final clustering results.

This modular architecture establishes inter-system biomechanical relationships, human-machine interaction logic, and spatial adaptation mechanisms while prioritizing functional realization. Apart from these, other sub-functions remain as independent functional modules. A more detailed illustration of the functional clustering process and results is shown in Fig. 6.

### Design and evaluation of the youth functional armchair solutions

#### Functional armchair solution design

**Fig. 7:**
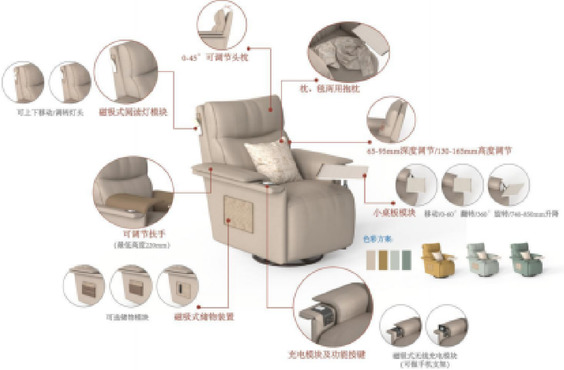
3D model rendering of solution 1.

**Fig. 8:**
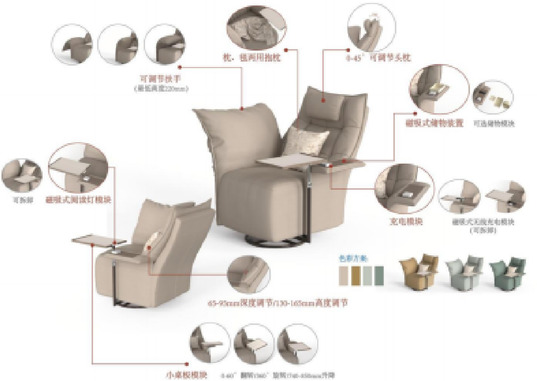
3D model rendering of solution 2.

Based on functional configuration requirements, user research feedback, and the functional module division strategy derived from previous research findings, two functional armchair solutions targeting young adults living alone were designed. Modeling was performed in Rhino 7 software, and rendering was completed in KeyShot 10 software. The 3D model renderings of solution 1 and solution 2 are shown in Figs. 7 and 8, respectively.

**Fig. 9:**
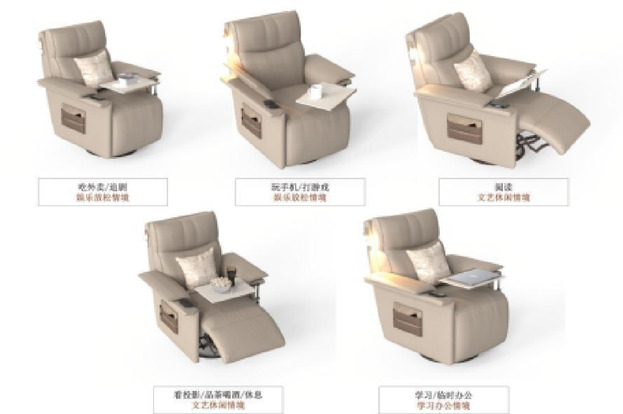
Scenario rendering of product usage in solution 1.

**Fig. 10:**
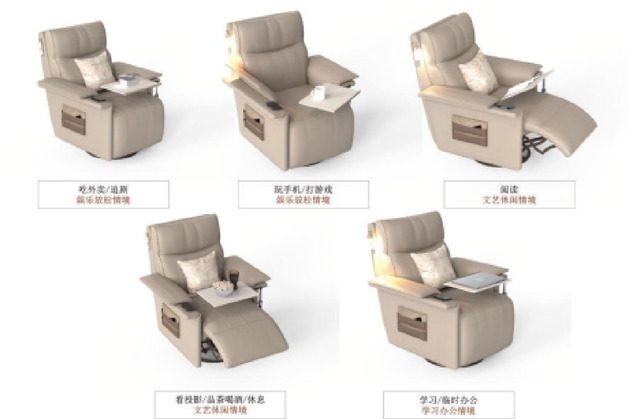
Scenario rendering of product usage in solution 2.

The product design adheres to the target scenario analysis requirements outlined in Section “Modular design”. The specific mapping relationships between user behaviors and corresponding scenarios for solution 1 and solution 2 are systematically presented in Figs. 9 and 10.

#### Evaluation of functional armchair design solutions

##### Establishing the design solution evaluation system

Based on user needs research, the findings were summarized and classified to construct an evaluation index system for the functional armchair. This resulted in an evaluation index system comprising four criterion layers and sub-criterion layers: Performance and Quality Indicators, Functional Indicators, Comfort Indicators, and User Experience Indicators, as detailed in Table 13.

**Table 13:** Evaluation index system.

Criterion layer	Sub-criterion layer	Evaluation criteria description
A1 Performance & Quality Indicators	B1 Safety	Whether the product is safe and stable for users and pets.
	B2 Durability	Whether materials and components are wear-resistant and durable, with a long service life.
	B3 Ease of Cleaning & Maintenance	Whether the product surface is easy to clean and whether it is stain-resistant.
	B4 Aesthetics	Whether the styling is aesthetically pleasing and whether color and material coordination is harmonious.
A2 Functional Indicators	B5 Functional Comprehensiveness	Whether the product covers a rich variety of functions.
	B6 Functional Practicality	Whether the functions effectively address genuine user needs.
	B7 Functional Rationality	Whether the functional design conforms to ergonomics and logic.
	B8 Functional Adaptability	Whether the functions adapt to different scenarios or postures.
A3 Comfort Indicators	B9 Support Comfort	Whether body parts fit well with the seat and receive effective support.
	B10 Tactile & Physical Comfort	Whether the fabric is skin-friendly, if material hardness is appropriate, and if it adapts to temperature/humidity conditions.
A4 User Experience Indicators	B11 Visual Comfort	Whether colors and appearance induce calm and relaxation, and if lighting design is soft.
	B12 Psychological Comfort	Whether use provides a sense of psychological security and pleasure.
	B13 Installation & Dismantling Convenience	Whether product assembly, disassembly, or module replacement is convenient.
	B14 Operational Convenience	Whether function adjustment and control are intuitive and efficient.
	B15 Ease of Use	Whether product use is automated and effortless for daily routines.
	B16 Humanization	Whether thoughtful details exist and if the design accommodates user habits.

##### Determining evaluation indicator weights and ranking using AHP

**Fig. 11:**
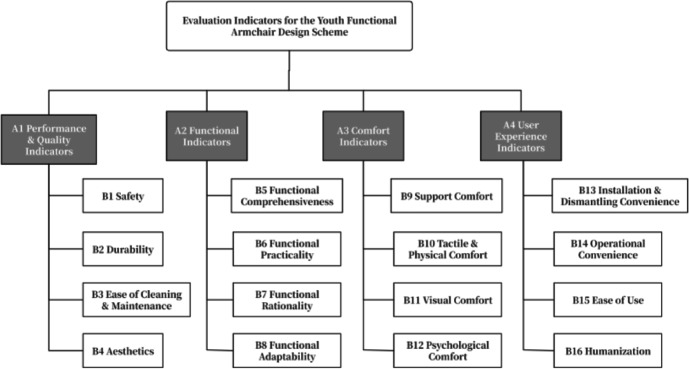
Hierarchical analysis model of the evaluation indicators.

An Analytic Hierarchy Process (AHP) model was constructed with the goal of evaluating functional armchair designs for young adults, including the target layer, four items for Criterion Layer A, and sixteen items for Sub-criterion Layer B, as illustrated in Fig. 11.

To ensure both professional relevance and user orientation, this study invited three furniture design experts and three target consumers to participate in the AHP evaluation. However, the limited sample size may restrict the generalizability of the derived weights. Overall, such a small purposive panel is more suitable for the comparative ranking and evaluation of alternative design concepts within a specific research context.

All judgment matrices passed the consistency check (CR < 0.1). Based on this, the weight values of the indicators in the sub-criterion layer were finalized. The comprehensive weights of each indicator are presented in Table 14. The ranking of the criterion layer weights is as follows: Comfort Indicators (0.3849) > Functional Indicators (0.3260) > Performance & Quality Indicators (0.1945) > User Experience Indicators (0.0946).

**Table 14:** Comprehensive weight table of evaluation indicators.

Criterion layer	Weight	Sub-criterion layer	Weight	Comprehensive weight	Ranking
A1 Performance & Quality Indicators	0.1945	B1 Safety	0.4905	0.0954	3
		B2 Durability	0.3120	0.0607	7
		B3 Ease of Cleaning & Maintenance	0.0893	0.0174	15
		B4 Aesthetics	0.1082	0.0210	12
A2 Functional Indicators	0.3260	B5 Functional Comprehensiveness	0.2088	0.0681	6
		B6 Functional Practicality	0.2760	0.0900	5
		B7 Functional Rationality	0.3728	0.1215	2
		B8 Functional Adaptability	0.1423	0.0464	10
A3 Comfort Indicators	0.3849	B9 Support Comfort	0.5220	0.2009	1
		B10 Tactile & Physical Comfort	0.2373	0.0913	4
		B11 Visual Comfort	0.1418	0.0546	8
		B12 Psychological Comfort	0.0989	0.0381	11
A4 User Experience Indicators	0.0946	B13 Installation & Dismantling Convenience	0.0693	0.0066	16
		B14 Operational Convenience	0.2222	0.0210	13
		B15 Ease of Use	0.4907	0.0464	9
		B16 Humanization	0.2179	0.0206	14

**Fig. 12:**
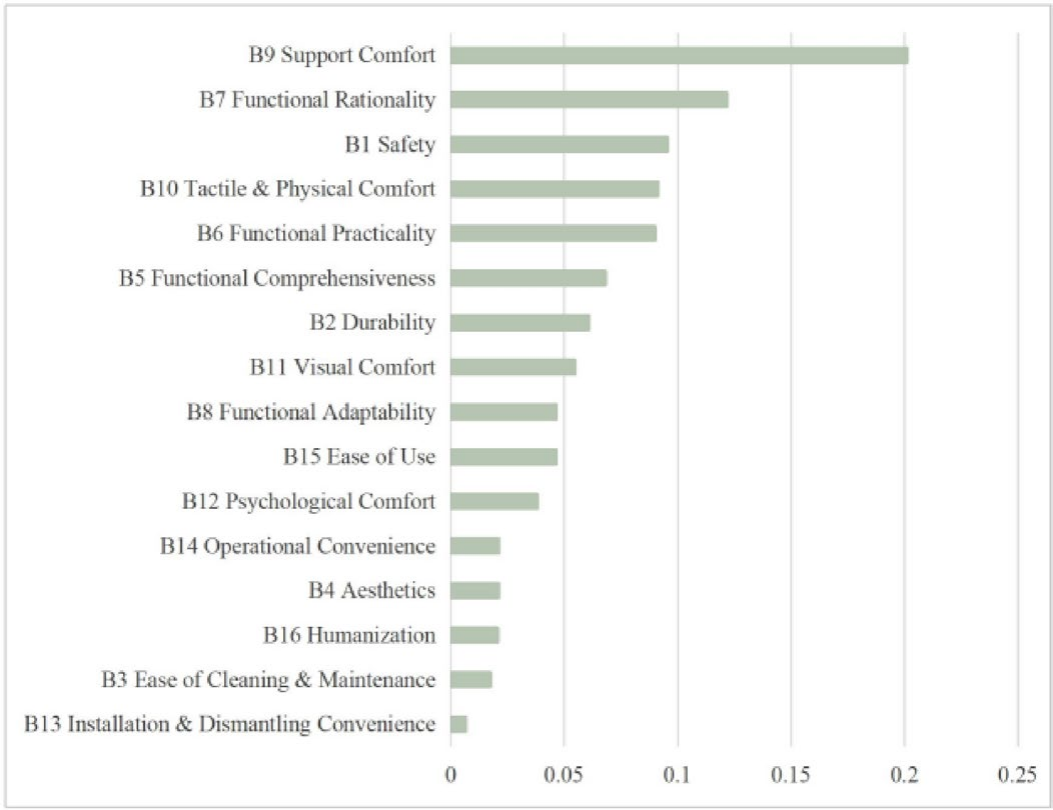
Comprehensive weight ranking.

The weight ranking within the sub-criterion layer (Fig. 12) shows that “Support Comfort” (0.2009), “Functional Rationality” (0.1215), and “Safety” (0.0954) are the three most critical indicators. This demonstrates that comfort, rationality of functional design, and product quality constitute the core dimensions for evaluating the superiority of functional armchairs.

##### TOPSIS evaluation of design solutions

**Fig. 13:**
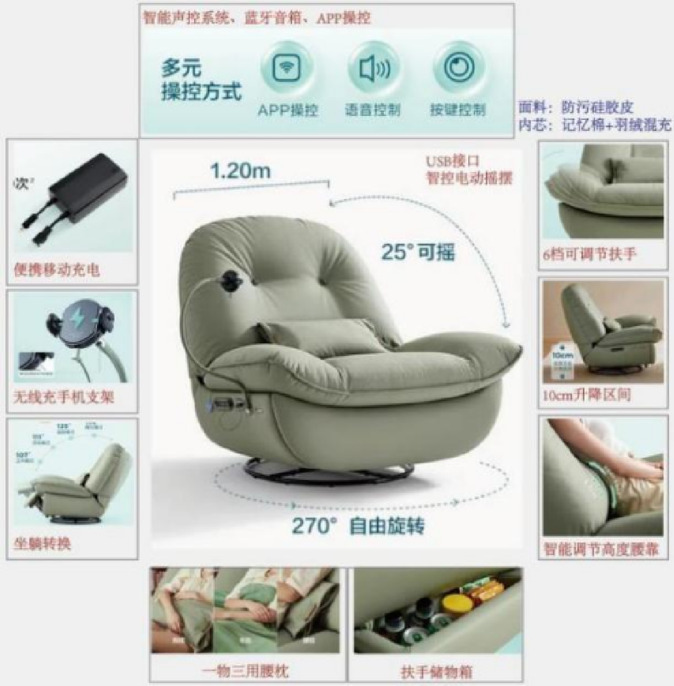
Functional armchair evaluation sample solution.

Six target consumers (comprising the three previously identified during the AHP scoring stage and three additional potential consumers) were invited to score the design solutions using a seven-point Likert scale. Prior to scoring, a focus group method was employed to select a functional armchair product that performed optimally across multiple dimensions—such as comprehensive functionality, materials, and form—as a benchmark sample for evaluation, as shown in Fig. 13.

Subsequently, the ratings for both the design solutions and the benchmark sample were averaged to form the initial evaluation matrix *F*. The specific results are presented in Table 15.

**Table 15:** Initial scoring matrix for the functional armchair solution of the function to be evaluated.

Evaluation indicator	Solution 1	Solution 2	Benchmark solution
fB1	6.3	6.0	5.3
fB2	5.7	6.0	5.7
fB3	6.2	6.5	5.2
fB4	5.7	6.5	6.3
fB5	5.7	5.8	5.7
fB6	6.0	6.7	4.0
fB7	5.5	6.5	5.7
fB8	6.3	6.8	4.2
fB9	6.0	6.8	4.3
fB10	4.8	4.8	4.5
fB11	5.3	6.0	6.0
fB12	5.5	6.5	5.2
fB13	6.3	6.5	5.0
fB14	6.5	6.7	6.7
fB15	6.2	6.7	5.5
fB16	5.8	6.7	4.3

The initial evaluation matrix was standardized, and the processing results are shown in Table 16.

**Table 16:** Standardization matrix for the functional armchair solution of the function to be evaluated.

Evaluation indicator	Solution 1	Solution 2	Benchmark solution
RB1	0.6184	0.5890	0.5203
RB2	0.5672	0.5971	0.5672
RB3	0.5973	0.6262	0.5010
RB4	0.5328	0.6076	0.5889
RB5	0.5740	0.5840	0.5740
RB6	0.6096	0.6807	0.4064
RB7	0.5368	0.6344	0.5563
RB8	0.6190	0.6682	0.4127
RB9	0.5978	0.6775	0.4284
RB10	0.5894	0.5894	0.5525
RB11	0.5298	0.5997	0.5997
RB12	0.5513	0.6515	0.5212
RB13	0.6092	0.6286	0.4835
RB14	0.5657	0.5831	0.5831
RB15	0.5818	0.6287	0.5161
RB16	0.5888	0.6802	0.4366

The standardized data were then subjected to weighted normalization using the weights derived from the AHP indicator weighting results. The outcomes of this weighted processing are presented in Table 17.

**Table 17:** Weighted matrix of functional armchair solution for evaluation function.

Evaluation indicator	Solution 1	Solution 2	Benchmark solution
uB1	0.0590	0.0562	0.0496
uB2	0.0344	0.0362	0.0344
uB3	0.0104	0.0109	0.0087
uB4	0.0112	0.0128	0.0124
uB5	0.0391	0.0398	0.0391
uB6	0.0549	0.0613	0.0366
uB7	0.0652	0.0771	0.0676
uB8	0.0287	0.0310	0.0191
uB9	0.1201	0.1361	0.0861
uB10	0.0538	0.0538	0.0504
uB11	0.0289	0.0327	0.0327
uB12	0.0210	0.0248	0.0199
uB13	0.0040	0.0041	0.0032
uB14	0.0119	0.0122	0.0122
uB15	0.0270	0.0292	0.0239
uB16	0.0121	0.0140	0.0090

The optimal and worst values of the indicators were identified as the positive and negative ideal solutions, respectively. These ideal solutions are listed in Table 18.

**Table 18:** Positive and negative ideal solutions of the solution to be evaluated.

Evaluation indicator	Positive ideal solution (A⁺)	Negative ideal solution (A–)
B1 Safety	0.0590	0.0496
B2 Durability	0.0362	0.0344
B3 Ease of Cleaning & Maintenance	0.0109	0.0087
B4 Aesthetics	0.0128	0.0112
B5 Functional Comprehensiveness	0.0398	0.0391
B6 Functional Practicality	0.0613	0.0366
B7 Functional Rationality	0.0771	0.0652
B8 Functional Adaptability	0.0310	0.0191
B9 Support Comfort	0.1361	0.0861
B10 Tactile & Physical Comfort	0.0538	0.0504
B11 Visual Comfort	0.0327	0.0289
B12 Psychological Comfort	0.0248	0.0199
B13 Installation & Dismantling Convenience	0.0041	0.0032
B14 Operational Convenience	0.0122	0.0119
B15 Ease of Use	0.0292	0.0239
B16 Humanization	0.0140	0.0090

The Euclidean distances from each functional armchair solution under evaluation to the positive and negative ideal solutions were calculated, along with their relative closeness degrees. These results were then ranked, as detailed in Table 19.

**Table 19:** Euclidean distance and relative closeness of the solution to be evaluated.

Solution	Positive ideal solution distance (S+)	Negative ideal solution distance (S–)	Relative closeness (C)	Ranking
Solution 1	0.0221	0.0413	0.6516	2
Solution 2	0.0028	0.0596	0.9551	1
Benchmark Solution	0.0594	0.0047	0.0728	3

The AHP–TOPSIS evaluation results indicate that Solution 2 is the optimal choice, as it achieved the highest relative closeness (Ci) value. Moreover, the Ci values of both design proposals were higher than that of the benchmark solution, suggesting that the proposed designs, developed on the basis of identified user needs, are better aligned with the target users in terms of both functionality and design. The evaluation combined expert and user judgments, adopted a unified seven-point scale, and used a benchmark product as a reference, which improved the comparability of the results. However, as the final ranking was based on a relatively small sample and lacked independent validation, the AHP–TOPSIS results should be regarded as preliminary. Future studies may further strengthen the findings through larger samples and external validation.

## Discussion

This study integrated the Kano model, modular design, AHP, and TOPSIS to develop a need-driven design framework for modular functional armchairs for young adults living alone. The main contribution of this framework lies not merely in combining multiple methods, but in establishing a sequential and interpretable design pathway that systematically links user-need identification, function screening, module generation, and solution evaluation. Unlike previous studies that often focused only on demand collection or qualitative descriptions of modularization, this study advances the design process toward a more operational and quantitative approach. More importantly, the framework clarifies how user needs can be progressively translated into product architecture and further into comparative design decisions, thereby improving the traceability of the overall design process.

Another important contribution of this study is that modularization is no longer treated simply as a strategy for structural decomposition, but is introduced as an intermediate decision-making layer connecting need priorities with product implementation logic. Through function–structure mapping, correlation assessment, and hierarchical clustering, prioritized user needs were further translated into 11 functional modules. This process improves the rationality and reproducibility of module partitioning, and also helps address a common contradiction in functional furniture design: the tendency to pursue excessive function stacking while neglecting personalization and practical usability.

The empirical results further support the validity of the proposed framework. The evaluation indicates that support comfort, functional rationality, and safety are the most important criteria in judging the quality of functional armchair design, suggesting that young adults living alone still regard core comfort and usability as fundamental. At the same time, the preference for technology-related and convenience-enhancing functions also reflects the lifestyle of this group. For young adults living alone, the armchair is no longer merely a seat for rest, but is increasingly becoming a compact activity hub for work, entertainment, device charging, reading, and temporary storage. Therefore, functions such as wireless charging, reading lights, modular tabletops, and storage are valued not because of their technological novelty, but because they enhance comfort in a more flexible and practical way.

In addition, the AHP-TOPSIS results show that Solution 2 achieved the highest relative closeness, and that both proposed design solutions outperformed the benchmark product. This indicates that the proposed framework can effectively improve the alignment between product solutions and user expectations, thereby enhancing design competitiveness.

At the same time, several limitations should be acknowledged. First, the process of determining weights in AHP inevitably involves a certain degree of evaluator subjectivity. Although all judgment matrices passed the consistency test, consistency alone cannot fully eliminate the influence of personal experience, disciplinary background, or professional preference on pairwise comparisons. In this study, the inclusion of both furniture design experts and target consumers enhanced the practical relevance of the evaluation; however, the resulting weights should still be understood as structured expert-informed judgments rather than fully objective measurements. Therefore, the findings of this study are better interpreted as case-based evidence for concept evaluation and methodological demonstration.

Second, the practical application of modular architecture in functional armchairs still faces several engineering challenges, especially at the level of modular connectors and interfaces. Repeated assembly and disassembly may affect the durability and stability of the connection structures, while the integration of electrical modules such as reading lights, charging units, and wireless power storage further increases the complexity of wiring arrangement, power supply safety, and maintenance. In addition, different modules may impose different requirements on structural tolerance, load-bearing performance, and installation precision. Therefore, although this study demonstrates the conceptual feasibility of need-driven module partitioning, future research should further verify the connector design, structural reliability, electrical safety, and long-term usability of modular interfaces through prototype testing and engineering experiments.

Future studies may also improve the robustness of the findings by expanding the evaluator sample size, increasing group diversity, conducting sensitivity analyses of indicator weights, and introducing additional validation procedures.

## Conclusion

This study developed a need-driven design framework for modular functional armchairs for young adults living alone. Rather than merely combining several established methods, the study contributes an interpretable design pathway that links user-need identification, functional prioritization, module generation, and solution evaluation. More specifically, it reconceptualizes modularization as an intermediate decision layer between user requirements and product implementation, thereby extending modular furniture design beyond qualitative decomposition toward a more structured and reproducible decision process.

The case study demonstrated the applicability of the framework. Fifteen core functions were identified and translated into 11 functional modules, and the evaluation results showed that support comfort, functional rationality, and safety were the most influential criteria. Among the alternatives, Solution 2 achieved the best overall performance, indicating that the proposed framework can improve the alignment between design solutions and user expectations.

The findings also have practical implications for the furniture industry. The proposed framework provides a useful reference for developing modular furniture that responds to limited living space, multifunctional domestic activities, and increasing demand for personalized home products. In this sense, the study offers not only an optimized armchair concept, but also a transferable methodological reference for the design of other furniture and durable consumer products with integrated functions.

This study has several limitations. First, the empirical investigation focused on a relatively limited sample of young adults living alone, which constrains the broader generalizability of the findings. Second, the evaluation stage relied to a considerable extent on expert judgment and small-sample user scoring, so the results should be interpreted as case-based comparative evidence rather than universally generalizable conclusions. Third, the validation was conducted mainly at the conceptual design level; engineering feasibility, production constraints, prototype testing, and long-term use performance were not fully examined.

Future research should include broader user samples and strengthen methodological robustness. Most importantly, the proposed module architecture should be further verified through physical prototyping, structural and engineering tests, and user evaluation, so that the balance among personalization, manufacturability, cost efficiency, and user experience can be examined more comprehensively.

## Supplementary Information

Below is the link to the electronic supplementary material.

Supplementary Material 1

## Data Availability

All data generated or analyzed during this study are included in this published article.
