# Supplementary material for: A need-driven design research of modular functional armchairs for young adults
Source: Sci Rep. 2026 Apr 29;16:13773. doi: 10.1038/s41598-026-49680-z (PMC13128914; doi:10.1038/s41598-026-49680-z)
Supplement: Supplementary file 1 — Supplementary Material 1 [file 41598_2026_49680_MOESM1_ESM.docx]

Appendix 1

Tab.6 Function directory

| **ID** | **Function** | **Sub-function** | **Technical Solution** | **Technical Principle** | **Case Example** |
| --- | --- | --- | --- | --- | --- |
| F1 | Sit-to-Lie Conversion | Multi-level Footrest/Backrest Adjustment | Electric Drive | The controller sends electrical signals to the motor, driving it to operate; the motor's power is transmitted to the seat's adjustment mechanism via a transmission device. | 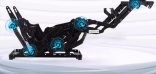 |
|  |  | Comfortable Support in Sitting/Lying Positions | 01.Temperature-Sensitive Memory Foam | Responds to body temperature, gradually softening as temperature rises while absorbing body pressure to adjust the body to the most comfortable posture. | 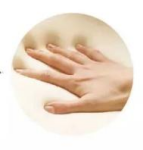 |
|  |  |  | 02. Active Contour Technology | Through real-time precise feedback from a pressure sensor system on posture, airbags in the sofa can automatically adjust the back, lumbar, and leg areas of the seat, achieving comfortable support with seamless contact. | 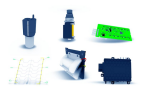 |
|  |  | Pet Anti-Pinch Function | Anti-Pinch Sensing Technology | Sensors detect resistance encountered during movement and transmit signals to the control system, which triggers appropriate protective measures (reverse movement or stop) immediately upon judgment. | 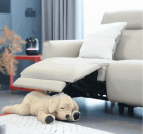 |
| F2 | Headrest Adjustment | Headrest Multi-level Adjustment | 01 Electric Drive Headrest Adjustment | The headrest motor drives the headrest adjustment via a transmission mechanism. | 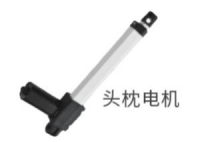 |
|  |  |  | 02 Pneumatic Headrest Adjustment | An electric pump or solenoid valve receives signals from the controller, adjusting the height, angle, and support force of the headrest by inflating/deflating airbags to change internal air pressure. | 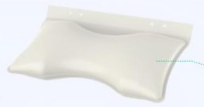 |
| F3 | Lumbar Support Adjustment | Lumbar Support Multi-level Adjustment | 01 Electric Drive Lumbar Support Adjustment | The lumbar support motor drives the lumbar support adjustment via a transmission mechanism. | 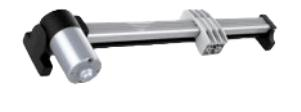 |
|  |  |  | 02 Pneumatic Lumbar Support Adjustment | An electric pump or solenoid valve receives signals from the controller, adjusting the height, angle, and support force of the lumbar support by inflating/deflating airbags to change internal air pressure. | 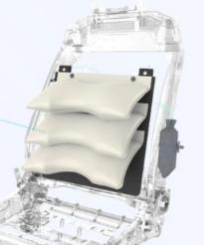 |
| F4 | Armrest Height Adjustment | Armrest Height Multi-level Adjustment | 01 Electric Drive Armrest Adjustment | The motor drives the armrest adjustment via a transmission mechanism. | 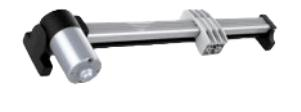 |
|  |  |  | 02 Elastic Mechanical Structure Adjustment | An elastic armrest frame is constructed using elastic materials and a mechanical structure, coupled with a locking device to achieve "deformation-locking-restoration" of the armrest, thereby adjusting its height. | 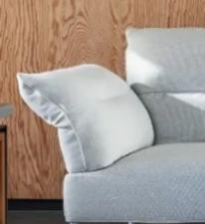 |
| F5 | Swivel Function | Rotation Lock | Base Turntable + Mechanical Locking | The rotating base swivels freely via bearings.  The locking mechanism typically includes components such as a locking lever, locking spring, and locking gear, used to lock the chair's swivel function. | 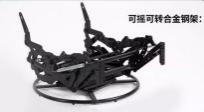 |
| F6 | Small Tabletop | Modular Connection | Traditional Mechanical Connection | Connectors such as clips and slots are used to attach the small tabletop to the functional sofa, enabling quick installation and removal. | 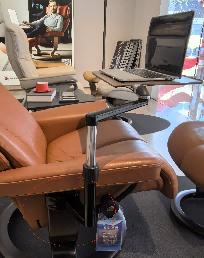 |
|  |  | Object Placement | Tabletop Panel | Items are stably supported via the tabletop surface. | 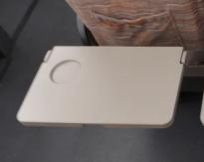 |
|  |  | Free Adjustment | 01 Mechanical Structure | Achieves free adjustment of the small tabletop via mechanical devices (lifting, telescoping, rotating, tilting mechanisms, etc.) | 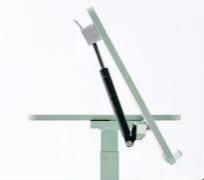 |
|  |  |  | 02 Robotic Arm | Controls the motion of each joint or axis through the robotic arm's control system to achieve movement, tilting, and rotation of the tabletop. | 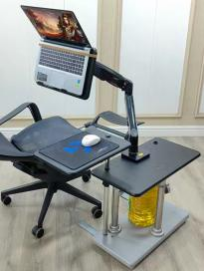 |
| F7 | Reading Light | Modular Connection | 01 Traditional Mechanical Connection | Connects the reading light to the main structure of the functional armchair via slots/clips or screws/nuts. | 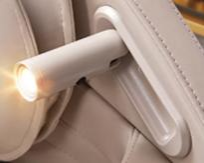 |
|  |  |  | 02 Magnetic Connection | Attaches the reading light to a magnetic base on the functional armchair using magnetic materials, powered by the armchair's built-in power system. | 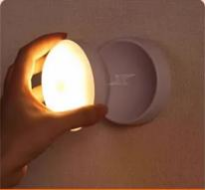 |
|  |  | Illumination | LED Light | Leverages the advantages of LED lights such as compact size, high brightness, and low energy consumption to provide illumination. | 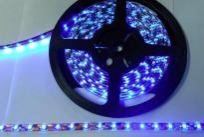 |
|  |  | Automatic Induction | Light Sensing + Human Presence Sensing | Detects ambient light levels and human proximity to provide illumination accordingly. | 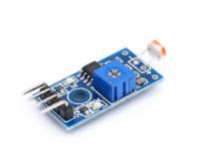 |
| F8 | Storage | Modular Connection | 01 Traditional Mechanical Connection | The storage module is designed as a detachable accessory, connected to the functional armchair via connectors (such as screws, clips, etc.). | 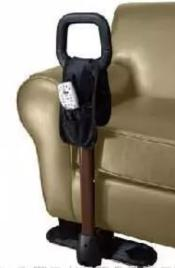 |
|  |  |  | 02 Magnetic Connection | Connects the sofa and the storage module via a magnetic device. | 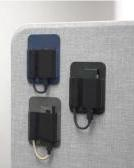 |
|  |  | Compartmentalized Storage | Designated Storage Areas | Divides different storage areas using partitions. | 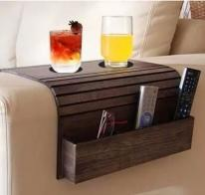 |
| F9 | Wireless Phone Charging | Modular Connection | Magnetic Connection | Connects the sofa and the wireless charging module via a magnetic device. | 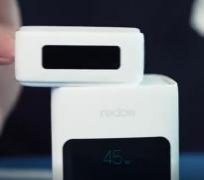 |
|  |  | Wireless Charging | Electromagnetic Induction Technology | Transmits electrical energy based on the principle of electromagnetic induction. | 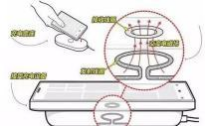 |
| F10 | Throw Pillow | Multi-functional Design | Multi-functional Design | Achieved through methods like folding and zipper fastening. | 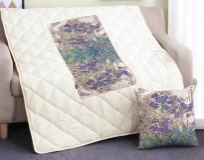 |
| F11 | Wireless Power Storage | Wireless Power Storage | Lithium Battery Power Storage | Utilizes dedicated lithium batteries for the functional armchair to store electrical power. | 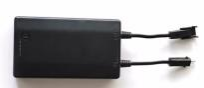 |
| F12 | Charging Interface | Charging via Interface | Power Transmission + Interface Conversion | The internal circuit system of the sofa converts the input AC power to a power specification suitable for the charging interface. | 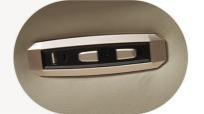 |
| F13 | Backrest Massage | Multi-level Massage Adjustment | 01 Mechanical Massage | Motor-driven robotic hands perform massage; massage intensity is adjusted by controlling the rotation speed and direction. | 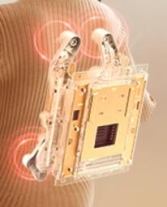 |
|  |  |  | 02 Pneumatic Massage | Massage is performed by applying pressure to the human body through inflation/deflation of airbags; massage intensity can be controlled by adjusting the air pump power and valve opening degree. | 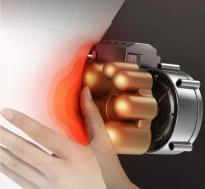 |
|  |  |  | 03 Vibration Massage | Mechanical vibrations generated by a vibration device produce a massage effect; massage intensity is adjusted by changing the frequency and amplitude of the vibration through regulating the motor's speed and power. | 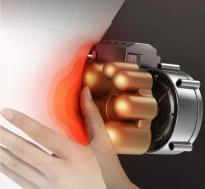 |
| F14 | Seat Cushion Heating | Multi-level Heating Adjustment | Built-in Heating Element | Converts electrical energy into thermal energy via an electric heating element, thereby raising the temperature of the seat cushion or massage area; temperature levels are controlled using components such as temperature sensors. | 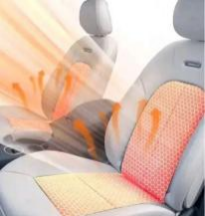 |
| F15 | Ventilation | Multi-level Adjustment | Built-in Fan Ventilation | Achieves ventilation by installing small fans inside the seat structure, utilizing their operation to create an airflow effect. | 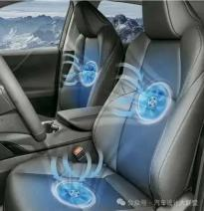 |

Appendix 2

Fig.7 Functional relevance matrix

|  | F_1_ | F_2_ | F_3_ | F_4_ | F_5_ | F_6_ | F_7_ | F_8_ | F_9_ | F_10_ | F_11_ | F_12_ | F_13_ | F_14_ | F_15_ | F_16_ | F_17_ | F_18_ | F_19_ | F_20_ | F_21_ | F_22_ | F_23_ |
| --- | --- | --- | --- | --- | --- | --- | --- | --- | --- | --- | --- | --- | --- | --- | --- | --- | --- | --- | --- | --- | --- | --- | --- |
| F_1_ | 1 |  |  |  |  |  |  |  |  |  |  |  |  |  |  |  |  |  |  |  |  |  |  |
| F_2_ |  | 1 |  |  |  |  |  |  |  |  |  |  |  |  |  |  |  |  |  |  |  |  |  |
| F_3_ |  |  | 1 |  |  |  |  |  |  |  |  |  |  |  |  |  |  |  |  |  |  |  |  |
| F_4_ |  | 0.5 |  | 1 |  |  |  |  |  |  |  |  |  |  |  |  |  |  |  |  |  |  |  |
| F_5_ |  | 0.5 |  |  | 1 |  |  |  |  |  |  |  |  |  |  |  |  |  |  |  |  |  |  |
| F_6_ |  |  |  |  |  | 1 |  |  |  |  |  |  |  |  |  |  |  |  |  |  |  |  |  |
| F_7_ |  |  |  |  |  |  | 1 |  |  |  |  |  |  |  |  |  |  |  |  |  |  |  |  |
| F_8_ |  |  |  |  |  |  | 0.5 | 1 |  |  |  |  |  |  |  |  |  |  |  |  |  |  |  |
| F_9_ |  |  |  |  |  |  |  | 0.75 | 1 |  |  |  |  |  |  |  |  |  |  |  |  |  |  |
| F_10_ |  |  |  |  |  |  |  | 0.75 | 0.75 | 1 |  |  |  |  |  |  |  |  |  |  |  |  |  |
| F_11_ |  |  |  |  |  |  |  |  | 0.5 |  | 1 |  |  |  |  |  |  |  |  |  |  |  |  |
| F_12_ |  |  |  |  |  |  |  |  |  |  | 0.75 | 1 |  |  |  |  |  |  |  |  |  |  |  |
| F_13_ |  |  |  |  |  |  |  |  |  |  | 0.75 | 0.75 | 1 |  |  |  |  |  |  |  |  |  |  |
| F_14_ |  |  |  |  |  |  |  |  |  |  |  |  |  | 1 |  |  |  |  |  |  |  |  |  |
| F_15_ |  |  |  |  |  |  |  |  |  |  |  |  |  | 0.75 | 1 |  |  |  |  |  |  |  |  |
| F_16_ |  |  |  |  |  |  |  |  |  |  |  |  |  | 0.75 | 0.5 | 1 |  |  |  |  |  |  |  |
| F_17_ |  |  |  |  |  |  |  |  |  |  |  |  |  |  |  | 0.75 | 1 |  |  |  |  |  |  |
| F_18_ |  |  |  |  |  |  |  |  |  |  |  |  |  |  |  |  |  | 1 |  |  |  |  |  |
| F_19_ |  |  |  |  |  |  |  |  |  |  |  |  |  |  |  |  |  |  | 1 |  |  |  |  |
| F_20_ |  |  |  |  |  |  |  |  |  |  |  |  |  |  |  |  | 0.5 |  |  | 1 |  |  |  |
| F_21_ |  | 0.5 |  |  |  |  |  |  |  |  |  |  |  |  |  |  |  |  |  |  | 1 |  |  |
| F_22_ |  |  |  |  |  |  |  |  |  |  |  |  |  |  |  |  |  |  |  |  |  | 1 |  |
| F_23_ |  |  |  |  |  |  |  |  |  |  |  |  |  |  |  |  |  |  |  |  | 0.5 | 0.5 | 1 |

Functional Structure Spatial Correlation Matrix

|  | F_1_ | F_2_ | F_3_ | F_4_ | F_5_ | F_6_ | F_7_ | F_8_ | F_9_ | F_10_ | F_11_ | F_12_ | F_13_ | F_14_ | F_15_ | F_16_ | F_17_ | F_18_ | F_19_ | F_20_ | F_21_ | F_22_ | F_23_ |
| --- | --- | --- | --- | --- | --- | --- | --- | --- | --- | --- | --- | --- | --- | --- | --- | --- | --- | --- | --- | --- | --- | --- | --- |
| F_1_ | 1 |  |  |  |  |  |  |  |  |  |  |  |  |  |  |  |  |  |  |  |  |  |  |
| F_2_ | 0.75 | 1 |  |  |  |  |  |  |  |  |  |  |  |  |  |  |  |  |  |  |  |  |  |
| F_3_ |  |  | 1 |  |  |  |  |  |  |  |  |  |  |  |  |  |  |  |  |  |  |  |  |
| F_4_ | 0.5 | 0.75 |  | 1 |  |  |  |  |  |  |  |  |  |  |  |  |  |  |  |  |  |  |  |
| F_5_ | 0.5 | 0.75 |  |  | 1 |  |  |  |  |  |  |  |  |  |  |  |  |  |  |  |  |  |  |
| F_6_ | 0.5 |  |  |  |  | 1 |  |  |  |  |  |  |  |  |  |  |  |  |  |  |  |  |  |
| F_7_ |  |  |  |  |  |  | 1 |  |  |  |  |  |  |  |  |  |  |  |  |  |  |  |  |
| F_8_ |  |  |  |  |  |  |  | 1 |  |  |  |  |  |  |  |  |  |  |  |  |  |  |  |
| F_9_ |  |  |  |  |  |  |  | 0.75 | 1 |  |  |  |  |  |  |  |  |  |  |  |  |  |  |
| F_10_ |  |  |  |  |  |  |  | 0.75 | 0.75 | 1 |  |  |  |  |  |  |  |  |  |  |  |  |  |
| F_11_ |  |  |  |  |  |  |  |  | 0.25 |  | 1 |  |  |  |  |  |  |  |  |  |  |  |  |
| F_12_ |  |  |  |  |  |  |  |  |  |  | 0.75 | 1 |  |  |  |  |  |  |  |  |  |  |  |
| F_13_ |  |  |  |  |  |  |  |  |  |  | 0.75 | 0.75 | 1 |  |  |  |  |  |  |  |  |  |  |
| F_14_ |  |  |  |  |  |  |  |  |  |  |  |  |  | 1 |  |  |  |  |  |  |  |  |  |
| F_15_ |  |  |  |  |  |  |  |  |  |  |  |  |  | 0.75 | 1 |  |  |  |  |  |  |  |  |
| F_16_ |  |  |  |  |  |  |  |  |  |  |  |  |  |  |  | 1 |  |  |  |  |  |  |  |
| F_17_ |  |  |  |  |  |  |  |  |  |  |  |  |  |  |  | 0.75 | 1 |  |  |  |  |  |  |
| F_18_ |  |  |  |  |  |  |  |  |  |  |  |  |  |  |  |  |  | 1 |  |  |  |  |  |
| F_19_ | 0.5 | 0.5 | 0.5 | 0.5 | 0.5 |  |  |  |  |  |  |  |  |  |  |  | 0.5 |  | 1 |  |  |  |  |
| F_20_ |  |  |  |  |  |  |  |  |  |  |  |  |  |  |  |  | 0.5 |  | 0.5 | 1 |  |  |  |
| F_21_ |  | 0.75 |  | 0.25 | 0.25 |  |  |  |  |  |  |  |  |  |  |  |  |  | 0.5 |  | 1 |  |  |
| F_22_ |  |  |  |  |  |  |  |  |  |  |  |  |  |  |  |  |  |  | 0.5 |  |  | 1 |  |
| F_23_ |  |  |  |  |  |  |  |  |  |  |  |  |  |  |  |  |  |  | 0.5 |  |  |  | 1 |

Functional Structure Distance Matrix D0

|  | F_1_ | F_2_ | F_3_ | F_4_ | F_5_ | F_6_ | F_7_ | F_8_ | F_9_ | F_10_ | F_11_ | F_12_ | F_13_ | F_14_ | F_15_ | F_16_ | F_17_ | F_18_ | F_19_ | F_20_ | F_21_ | F_22_ | F_23_ |
| --- | --- | --- | --- | --- | --- | --- | --- | --- | --- | --- | --- | --- | --- | --- | --- | --- | --- | --- | --- | --- | --- | --- | --- |
| F_1_ | 1 |  |  |  |  |  |  |  |  |  |  |  |  |  |  |  |  |  |  |  |  |  |  |
| F_2_ | 0.694 | 1 |  |  |  |  |  |  |  |  |  |  |  |  |  |  |  |  |  |  |  |  |  |
| F_3_ |  |  | 1 |  |  |  |  |  |  |  |  |  |  |  |  |  |  |  |  |  |  |  |  |
| F_4_ | 0.796 | 0.398 |  | 1 |  |  |  |  |  |  |  |  |  |  |  |  |  |  |  |  |  |  |  |
| F_5_ | 0.796 | 0.398 |  | 0.898 | 1 |  |  |  |  |  |  |  |  |  |  |  |  |  |  |  |  |  |  |
| F_6_ | 0.796 |  |  |  |  | 1 |  |  |  |  |  |  |  |  |  |  |  |  |  |  |  |  |  |
| F_7_ |  |  |  |  |  |  | 1 |  |  |  |  |  |  |  |  |  |  |  |  |  |  |  |  |
| F_8_ |  |  |  |  |  |  | 0.704 | 1 |  |  |  |  |  |  |  |  |  |  |  |  |  |  |  |
| F_9_ |  |  |  |  |  |  |  | 0.25 | 1 |  |  |  |  |  |  |  |  |  |  |  |  |  |  |
| F_10_ |  |  |  |  |  |  |  | 0.25 | 0.25 | 1 |  |  |  |  |  |  |  |  |  |  |  |  |  |
| F_11_ |  |  |  |  |  |  |  |  | 0.602 |  | 1 |  |  |  |  |  |  |  |  |  |  |  |  |
| F_12_ |  |  |  |  |  |  |  |  |  |  | 0.25 | 1 |  |  |  |  |  |  |  |  |  |  |  |
| F_13_ |  |  |  |  |  |  |  |  |  |  | 0.25 | 0.25 | 1 |  |  |  |  |  |  |  |  |  |  |
| F_14_ |  |  |  |  |  |  |  |  |  |  |  |  |  | 1 |  |  |  |  |  |  |  |  |  |
| F_15_ |  |  |  |  |  |  |  |  |  |  |  |  |  | 0.25 | 1 |  |  |  |  |  |  |  |  |
| F_16_ |  |  |  |  |  |  |  |  |  |  |  |  |  | 0.556 | 0.704 | 1 |  |  |  |  |  |  |  |
| F_17_ |  |  |  |  |  |  |  |  |  |  |  |  |  |  |  | 0.25 | 1 |  |  |  |  |  |  |
| F_18_ |  |  |  |  |  |  |  |  |  |  |  |  |  |  |  |  |  | 1 |  |  |  |  |  |
| F_19_ | 0.796 | 0.796 | 0.796 | 0.796 | 0.796 |  |  |  |  |  |  | 0.796 |  |  |  |  | 0.796 |  | 1 |  |  |  |  |
| F_20_ |  |  |  |  |  |  |  |  |  |  |  |  |  |  |  |  | 0.5 |  | 0.796 | 1 |  |  |  |
| F_21_ |  | 0398 |  | 0.898 | 0.898 |  |  |  |  |  |  |  |  |  |  |  |  |  | 0.796 |  | 1 |  |  |
| F_22_ |  |  |  |  |  |  |  |  |  |  |  |  |  |  |  |  |  |  | 0.796 |  |  | 1 |  |
| F_23_ |  |  |  |  |  |  |  |  |  |  |  |  |  |  |  |  |  |  | 0.796 |  | 0.704 | 0.704 | 1 |
